# Expression of apolipoproteins L in neutrophils during sepsis

**DOI:** 10.1186/cc14115

**Published:** 2015-03-16

**Authors:** I Akl, C Lelubre, M Piagnerelli, P Biston, P Uzureau, H Fayyad Kazan, B Badran, M Ezzedine, K Zouaoui Boudjeltia, L Vanhamme

**Affiliations:** 1 CHU de Charleroi-Hopital Andre Vesale, Montigny-Le-Tilleul, Belgium; 2 Institut Jules Bordet, Université Libre de Bruxelles, Belgium; 3 Doctoral School of Sciences and Technology, Platform of Research and Environmental Sciences, Beirut, Lebanon; 4 Institute of Medicine and Molecular Biology IBMM, Charleroi, Belgium

## Introduction

Sepsis is characterized by a strong systemic inflammatory reaction. The pathogenesis is driven by alterations in the immune system and is associated with high neutrophil counts related to a specific delay in apoptosis [1]. The apolipoproteins L (ApoLs) family comprises six members in humans (ApoL1 to ApoL6). In light of their deregulated expression in several pathologies, they are likely to be important molecular players of programmed cell death [2]. We analyzed ApoL expression in cohorts of septic and nonseptic ICU patients and healthy volunteers in order to test whether ApoLs could be involved in the neutrophil apoptotic program.

## Methods

By means of magnetic cell sorting, peripheral neutrophils were purified from 20 healthy volunteers and 40 ICU patients with (*n *= 20) or without sepsis (*n *= 20). ApoL expression was analyzed at the mRNA and protein levels by real-time PCR and western blot analysis respectively. Apoptosis of purified neutrophils was assessed using flow cytometry following 4 and 24 hours of *in vitro *incubation. We monitored the expression of C-reactive protein (CRP), an inflammatory marker, and its correlation with ApoL expression in PMNs was studied by linear regression analysis.

## Results

Our results showed a significant downregulation in mRNA expression of ApoL1 (*P *< 0.0001), ApoL2 (*P *= 0.0009), ApoL3 (*P *< 0.0001) and ApoL6 (*P *= 0.0003) in purified PMNs from ICU patients as compared with the healthy individuals. This downregulation was also validated at the protein level for ApoL1 and ApoL2, whereas ApoL 6 was upregulated in septic patients. We could not detect ApoL3 protein in any of the cohorts. This was accompanied by a significant delay in PMN apoptosis in septic patients as compared with healthy volunteers (*P *< 0.05) at 4 and 24 hours. We also showed a strong negative correlation in the three mixed groups between CRP and ApoL1 (*R *= -0.607), ApoL2 (*R *= -0.651), ApoL3 (*R *= -0.578) and ApoL6 (*R *= -0.506).

## Conclusion

The altered apoptotic fate of neutrophils in sepsis was correlated with the modification of the expression profile of ApoLs, a family of proteins thought to be involved in the apoptotic process. The role of these proteins in the sepsis-associated phenotype of neutrophils remains to be further elucidated.
